# The Impact of Rainfall Variability on Diets and Undernutrition of Young Children in Rural Burkina Faso

**DOI:** 10.3389/fpubh.2021.693281

**Published:** 2021-09-20

**Authors:** Isabel Mank, Kristine Belesova, Jan Bliefernicht, Issouf Traoré, Paul Wilkinson, Ina Danquah, Rainer Sauerborn

**Affiliations:** ^1^Heidelberg Institute of Global Health (HIGH), Faculty of Medicine and University Hospital, Heidelberg University, Heidelberg, Germany; ^2^Department of Public Health, Environments and Society and Centre on Climate Change and Planetary Health, London School of Hygiene and Tropical Medicine (LSHTM), London, United Kingdom; ^3^Institute of Geography, Faculty of Applied Computer Science, University of Augsburg, Augsburg, Germany; ^4^Centre de Recherche en Santé de Nouna (CRSN), Institut National de Santé Publique, Nouna, Burkina Faso; ^5^Institut Universitaire de Formations Initiale et Continue (IUFIC), Université Thomas Sankara (UTS), Ouagadougou, Burkina Faso

**Keywords:** dietary patterns, reduced rank regression, West Africa, precipitation, climate change, child undernutrition

## Abstract

**Background:** Climate change and consequent increases in rainfall variability may have negative consequences for the food production of subsistence farmers in West Africa with adverse impacts on nutrition and health. We explored the pathway from rainfall through diet up to child undernutrition for rural Burkina Faso.

**Methods:** The study used data of a dynamic cohort with 1,439 children aged 7–60 months from the Nouna Health and Demographic Surveillance Site (HDSS) for 2017 to 2019. We assessed data on diets, height, weight, household characteristics, and daily precipitation (from 1981 to 2019). Principal component analysis was used to identify distinct child dietary patterns (Dietary Pattern Scores, DPS). These were related to 15 rainfall indicators by area to obtain a precipitation variability score (PVS) through reduced rank regression (RRR). Associations between the PVS and anthropometric measures, height-for-age (HAZ), and weight-for-height (WHZ), were examined using multi-level regression analysis.

**Results:** Stunting (HAZ < −2) and wasting (WHZ < −2) were seen in 24 and 6% of the children. Three main dietary patterns were identified (market-based, vegetable-based, and legume-based diets) and showed mixed evidence for associations with child undernutrition. The RRR-derived PVS explained 14% of the total variance in these DPS. The PVS was characterized by more consecutive dry days during the rainy season, higher cumulative rainfall in July and more extremely wet days. A 1-point increase in the PVS was associated with a reduction of 0.029 (95% CI: −0.06, 0.00, *p* < 0.05) in HAZ in the unadjusted, and an increase by 0.032 (95% CI: 0.01, 0.06, *p* < 0.05) in WHZ in the fully adjusted model.

**Conclusion:** Rainfall variability was associated with dietary patterns in young children of a rural population of Burkina Faso. Increased rainfall variability was associated with an increase in chronic undernutrition, but not in acute undernutrition among young children.

## Introduction

Climate change drives increased weather variability and intensity such as by extreme rainfall events and mini-droughts during the rainy season. These changes have become a constant hazard that threaten to amplify existing risks to health and nutrition (1–3). Climate change is likely to increase health inequities, disproportionally affecting the most vulnerable and disadvantaged populations living under environmental pressure and having the least resources for adaptation and mitigation (1, 4, 5).

Despite the increasing awareness of the effects of climate on public health (1, 6), climate change simulations still bear large uncertainties for many regions of the world such as for West Africa (1, 2, 7–9). So far, West Africa is characterized by a large decadal precipitation variability (10, 11) and intensity of rainfall and drought events (1, 7, 12, 13). While the 1970s and 1980s were marked by a long-lasting drought period in the Sahel region, some recovery of the rainfall including increased precipitation extremes (14, 15) in combination with an increase toward more extreme temperatures were observed in the last decades (2, 7, 8, 16). Those climatic changes can have severe negative implications on agricultural production and food availability, and thus, lead to food insecurity and undernutrition in this region (2, 9, 17).

West Africa is also characterized by rural subsistence farming with little agricultural inputs and technology. Subsistence farmers, therefore, rely heavily on rainfall for agricultural production for their survival, financial income and family nutrition (18–20). Thus, if agricultural yields decline or fail, food stocks get empty before the next harvest and food prices will rise causing the families to adapt their food sources and diets (21–23). Diets can be assessed through dietary patterns, which reflect the complexity of a diet and provide a realistic impression of the overall diet structure (20, 24, 25). Children aged <5 years specifically need sufficient food and nutrients for their development and growth, wherefore a lack manifests in a higher risk for stunting, wasting, impeded cognitive development and subsequent death (17, 26, 27), which starts already *in utero*, continues into early childhood and manifests in adulthood (3, 5, 28–31). Both nutrition and health of children are assumed to worsen with the impacts of climate change and may even hamper efforts taken to reduce undernutrition in the coming decades (32, 33).

Yet, studies looking at associations between climate change and undernutrition among children aged <5 years are neither vast nor comparable due to a lack of robust data and substantial heterogeneity of research methods (17, 34). In this study, we explored how rainfall variability (by location) is linked through diets to child undernutrition in rural Burkina Faso. Although the study region in the West African country experienced a great reduction in child stunting and wasting from 45 and 26% in 1999 (35) to 26 and 7% in 2018 (20), the numbers are still high and threaten to rise again due to climate change (36). We sought to explore the hypotheses that (i) rainfall variability is associated with the composition of the children's diet, and that (ii) rainfall variability related to the children's diet is associated with their nutritional status. Rainfall variability was chosen because of its high relevance for food production in the study area (2). In contrast, temperature variability mainly confers direct impacts on physical health through heat stress (37, 38).

## Materials and Methods

### Study Site and Population

The study was conducted in the Nouna Health and Demographic Surveillance System (HDSS) area in rural North-Western Burkina Faso, a land-locked West African country. The Nouna HDSS exists since 1992 and includes 59 villages with over 107,000 inhabitants (by 2017), who have been part of a population under continuous health and demographic surveillance (39). Small-scale subsistence farming is the main type of livelihood (19). Maize, sorghum, millet and rice are the main stable crops consumed by children <5 years of age in the area. In the study region, stunting (26%), and wasting (7%) are common among children aged <5 years (20, 40, 41), where also mortality from undernutrition continuous to be high (17). We had preliminary evidence of spatial variations in health outcomes and rainfall variability in the HDSS area (42), wherefore we aimed to explore this difference combining available health and rainfall data by small spatial scale.

### Study Design

**Figure 1:**
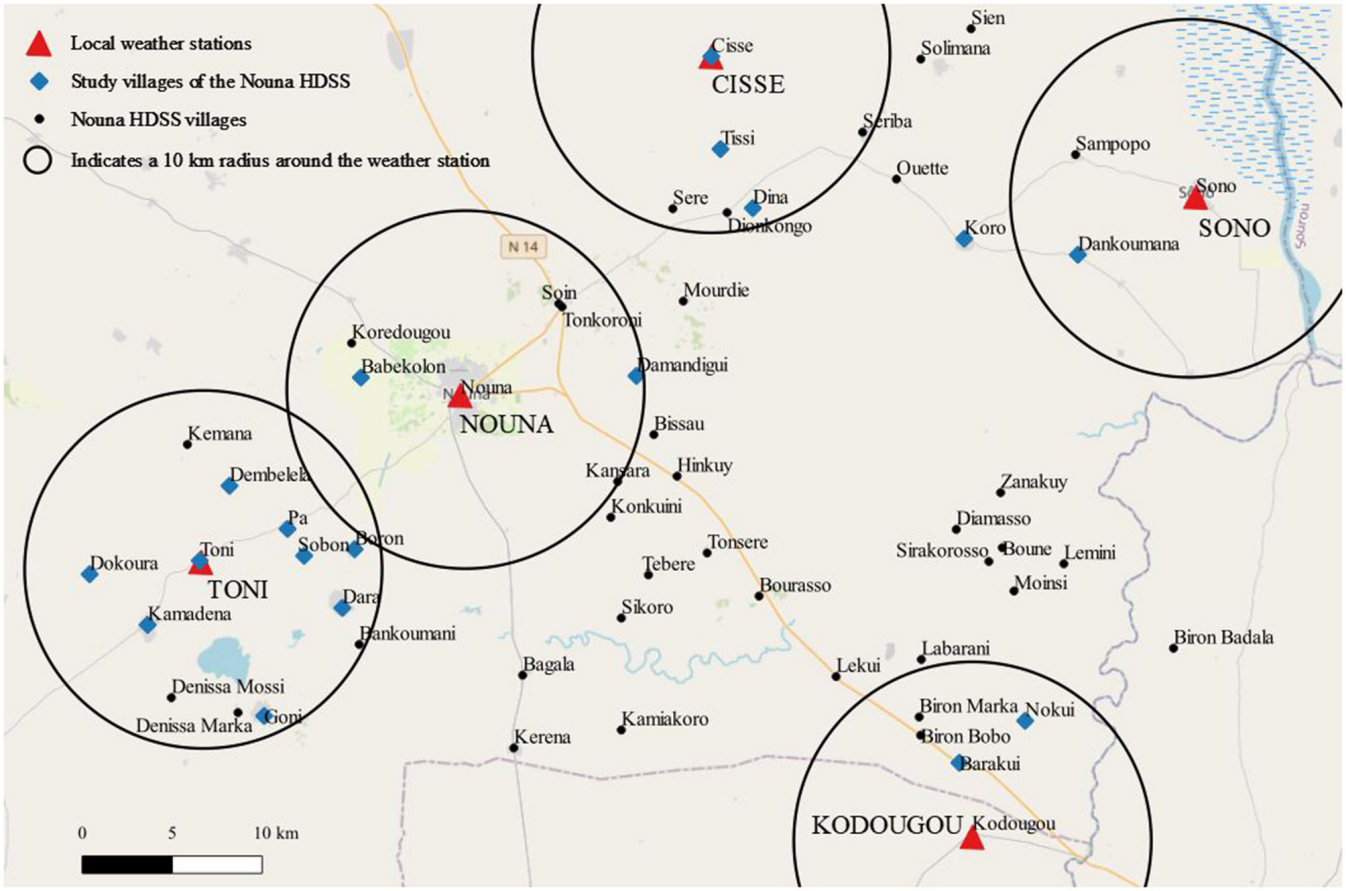
Study area with five strata in the Nouna HDSS area. One village (Koro) was added in order to assure the representativeness of the less densely populated stratum although it lies a few kilometers outside of the sampling frame. The circles indicate the 10 km radius around the respective weather station.

The study was designed as a dynamic cohort comprising 1,439 children aged 7–60 months, an age group when breastfeeding is complemented by soft and solid foods. We selected the participants using a two-stage stratified random sampling approach. In the first stage, 32 villages situated within a 10 km radius around five local weather stations (stratum) were considered for inclusion. Figure 1 depicts the location of the five strata: Cissé (−3.736°E/12.896°N), Sono (−3.494°E/12.828°N), Kodougou (−3.605°E/12.516°N), Toni (−3.991°E/12.650°N), and Nouna (−3.861°E/12.731°N). The number of study villages was then selected based on proportionality-to-population size within each stratum, which led to a total of 18 villages.

In the second stage, we defined as sampling unit all households with at least one child between 7 and 60 months, using data of the most recent HDSS data collection from early 2017. Households were defined as independent socio-economic units living in the same compound and joining resources to meet basic dietary and other vital needs (43). Based on population density of each stratum, the number of children was then defined and the children randomly selected. Data were collected each year from August through September from 2017 to 2019. Due to the study design, children, who had reached their 5th birthday, were randomly replaced by the same number of children aged 7–23 months to ensure a fairly constant proportion of children in each age group: 7–23, 24–35, 36–47, and 48–60 months. Households selected to take part, but who did not wish to give consent, were replaced by another household with a child of a similar age in the same village.

### Outcome and Exposure Measures

#### Rainfall Measurements

Daily rainfall was derived from a combination of on the ground and satellite observations. On the ground data was obtained from the Agence Nationale de la Météorologie (ANAM) in Burkina Faso and belonged to a novel quality-controlled precipitation database established as part of the WASCAL (West African Science Service Center on Climate Change and Adapted Land Use) observation network (39, 44). The on the ground-rainfall dataset was based on daily measurements from 19 rainfall stations located in the Nouna HDSS area surroundings (<80 km) and included data from 1981 to 2016. Then, a stochastic resampling method was used to generate daily time series for the required time period (2016 to 2019) for the centers of the five strata and which allowed for a statistical correction of the Climate Hazards Group Infrared Precipitation with Stations (CHIRPS) dataset using quantile-mapping (45, 46).

The gridded satellite rainfall product CHIRPS is advantageous to other satellite data as it has a high spatial resolution of 0.05° and has been explicitly designed to support drought analysis in food insecure regions (47). Dembélé and Zwart (48) showed that CHIRPS outperform other satellite products for Burkina Faso.

#### Anthropometric Measures of Undernutrition

Anthropometric measurements of children were taken twice by trained local field agents. The mean of the measurements was used for analysis (20). Recumbent length (Seca 417) and standing height using a stadiometer (Seca 213) were taken to the nearest cm. Weight was measured using tared weighing scales to the nearest 100 g (Seca 878) following WHO standards (49–51). Anthropometric data were analyzed using the WHO Child Growth Standards R igrowup package to derive deviations of weight-for-height (WHZ) and height-for-age (HAZ) of the children by sex from the distribution of the WHO reference population of children (50). Each child was assigned a respective z-score and considered stunted or wasted at < -2 (50, 52).

#### Dietary Assessment

A culturally adapted, semi-quantitative Food Frequency Questionnaire (FFQ) was administered by the same trained field agents, who took the anthropometric measurements. The dietary assessment tools and procedures have been described in detail in Mank et al. (20). In short, the FFQ assessed the child's food intake frequency over the preceding 7 days during the lean and rainy season. The lean season occurs from June to mid-September, the rainy season takes place from May to November (53). The data collection took place in August and September and thus, just before the first harvest. The field agents talked directly to the mother or caregiver of the child in the local language (mostly Dioula or Moré). The recall took on average 30 min. The administered FFQ listed 117 locally available food items that were commonly consumed by children in the Nouna HDSS area and included information on the intake frequencies expressed as number of servings per week. We did not assess food quantities. The included food items were selected based on local observations and previous studies (54, 55) as well as finalized during extensive training sessions with the local field agents.

#### Covariates of Child Undernutrition

Field workers were also trained in conducting household interviews in the local languages. The household questionnaire captured information that are commonly associated with child undernutrition (56, 57). Those include socio-demographic and -economic factors (mothers' and household heads' education and ethnicity, household wealth, siblings <5 years, and currently breastfeeding) and clinical indicators of the child (history of diarrhea and fever over the previous 2 weeks) (28, 40). Household wealth was calculated using the international wealth index (IWI) (58), which is internationally comparable and does not rely on expenditure information and price values. The IWI value was divided into quintiles based on ownership of selected household assets with 1 being the poorest and 5 being the wealthiest quintile. Details on the construction of the IWI for the Nouna HDSS area can be found in Mank et al. (20).

### Data Management and Statistical Methods

#### Data Management and Handling of Missing Data

The paper-based data was entered by two research assistants into EpiInfo version 3.5.3 and checked for correctness by a third person in case of incongruent entries. Data quality checks, data cleaning and statistical analyses were performed with StataIC version 15.0 and R version 3.4.3. Anthropometric measurements were excluded from the analyses in case of implausible birth dates, if the difference of the two measurements per child was too high (>2 cm for height and >0.9 kg for weight) or if computed *z*-scores were biologically impossible as defined by the WHO (59). Due to missing values for socio-demographic data, 78 observations were later excluded for the regression analysis from the original 1,439 observations. This led to a total of 1,364 observations for HAZ data and 1,359 observations for WHZ data.

#### General Characteristics

Demographic, socio-economic, clinical, and anthropometric characteristics are presented for the total study population (*N* = 1,439). We display absolute numbers and proportions for categorical variables, and medians and interquartile ranges (IQR) for continuous variables.

#### Rainfall Indicators

The daily precipitation data from 1981 to 2019 was prepared according to 15 precipitation indicators by the five strata (Supplementary Table S1). These indicators allow measuring rainfall variability and climate extreme events. Out of the 15 precipitation indicators, nine were derived from the Expert Team from Climate Change Detection and Indices (ETCCDI) (http://etccdi.pacificclimate.org/). Those indices were calculated using the R software package “RClimDex (1.0)” (60). Two indicators were added providing indication of the length of the wet season and the maximum number of consecutive dry days (RR < 1 mm) during the wet season, also called mini-droughts, and slightly adapted based on De Longueville et al. (12). The start of the wet season was defined as any date after 1st May, when two times >10 mm rain fell within 14 days, and the end of the wet season was defined as any date after 1st September, when 0 mm rain fell over 15 days. Lastly, four more indicators were added based on West et al. (61), which are considered as essential for crop growth in the study area: the number of days of so-called “big rains” (*R* > 20 mm) and the amount of rain falling in each month of July and August [also (2)].

#### Dietary Pattern Scores

Dietary pattern scores (DPS) were generated from the FFQ through principle component analysis (PCA). We generated 30 food groups from 117 food items based on their similarities in nutrient profile and culinary use (24, 62). Food items were excluded from the PCA, when they were never consumed by more than 96% of the children. PCA is a dimension-reduction technique that uses the correlation structure of intake frequencies to identify underlying food combinations commonly consumed by the target group (62, 63). This approach allowed us to make full use of the correlation structure of the intake frequencies of 30 food groups. Such a detail in diet data is unusual and unique for such a study setting and population group. We believe that the data-driven approach is an advantage over a priori approaches, because it goes beyond the simple quality indices to assess the nature of the diets in this study population. Information on the construction of the food groups and the method applied are detailed in Mank et al. (20), where dietary patterns were created with the 2018 data of the same study population.

**Figure 2:**
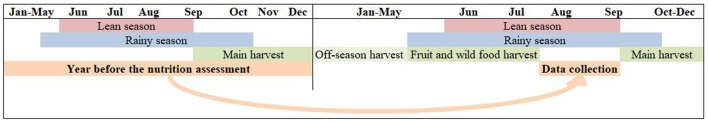
Description of the study hypothesis based on the seasonal calendar of Burkina Faso. (adapted from Dixon & Holt (53)).

For this analysis, food item information was pooled from 2017 to 2019 assuming similarity in dietary patterns between years as our data collection took place during the same season. We made this assumption for three reasons: (i) In a subsistence farming environment such as in our study region, households rely on the foods they harvest, which are led by the seasonal calendar (Figure 2) and not markets. Thus, (ii) the food market in the region is still very much locally oriented with little imports from other countries that would allow a variability of foods. In addition, (iii) dietary patterns reflect the combination of food items commonly consumed together, but not the diversity or quantity of foods. The combination of food items consumed together does not change as much as the food items consumed individually. Yet, variability in diets between seasons within the same year could be the case (64).

For the generation of DPS, an Eigenvalue criterion of >1.5 was applied and food groups with factor loadings of ≥|0.40| were considered as major contributors to the dietary pattern. Each child received a factor score for each dietary pattern based on reported intake frequencies of this food group. The factor score represents the adherence of each child to the respective dietary pattern.

#### Construction of a Precipitation Variability Score

The selected precipitation indicators were standardized to *z*-scores, representing the deviation of the annual precipitation data of each study year (2016–2019) from the reference rainfall data (1981–2019) by stratum. The higher the *z*-score, the greater is its annual variability (Supplementary Table S2). For the present analysis, we hypothesized that rainfall variability is associated with diets and so stunting and wasting with a time-delay; precisely in the year before the nutrition assessment (log-1) (Figure 2). Thus, the harvest from October to December impacts the diets of the following months and thus the nutritional status by August/September the next year, when the population data was collected.

In order to calculate the direct impact of rainfall on dietary patterns, we applied a reduced rank regression (RRR) analysis as a dimension reduction technique to create a precipitation variability score (PVS) (65, 66). This technique combines exploratory and hypothesis-based approaches for the identification of latent risk factors and is purely data-driven. We chose a data-driven approach because (i) there is limited knowledge on the associations of weather variables with child diet and nutritional status, and because (ii) weather or rainfall can be defined by more than only cumulative rainfall. We elaborate on both arguments in the discussion. Through the use of RRR, we were able to discover a set of precipitation indicators (predictor variables) that (i) commonly occur together and (ii) explain the most variation in the PCA-derived DPS (response variable). The RRR method was applied in the SAS software (SAS Institute, Inc., Cary, North Carolina). Only the first pattern score was extracted, which explained the highest variance in the response variables. Each child was matched with its PVS. Precipitation indicators with factor loadings of ≥|0.20| were considered to be major contributors to the PVS. The relationships of the PVS with the 15 precipitation indicators were described with unadjusted coefficients and partial coefficients adjusted for age and sex of the child and stratum using the parametric Pearson correlation coefficients.

#### Associations Between Rainfall Variability and Child Undernutrition

In order to establish the associations between the PVS and child undernutrition, multi-level mixed effects regression models were constructed and accounted for random effects at three levels: the village-, the household- and the child-level. We calculated regression-coefficients and their 95% confidence intervals (CIs) for HAZ and WHZ across tertiles of the PVS, assigning the first tertile as the reference category and constructed three models to assess the role of mediating factors. The unadjusted model presents associations of PVS with HAZ and WHZ, respectively; Model 1 was adjusted for age and sex of the child; and Model 2 was additionally adjusted for socio-economic factors and health indicators. Furthermore, we modeled the PVS associations per 1-point increase of the PVS presenting the difference in means of HAZ and WHZ. A 1-point increase in the PVS translates into an increased deviation of the rainfall pattern away from the norm.

## Results

### Study Population and Rainfall Characteristics

The study sample comprised 1,439 children between 7 and 60 months of age of which 52% were girls. The prevalence of stunting (HAZ < −2) and wasting (WHZ < −2) was 24 and 6% combined for the study period 2017–2019. Characteristics of the study sample can be found in Table 1. In short, mothers reported that 32% of the children had fever and 16% had diarrhea over the previous 2 weeks. Twenty-six percent of the mothers breastfed their child at the time of data collection (age when stopped breastfeeding was at median 24 ± 0 months of age). Independent of age, 97% of the children consumed cereals, roots and tubers, 93% vitamin-A rich leaves, 68% fruits, and 62% vegetables over the previous 7-days during the lean season in the study years. Illiteracy was high with 79% of the mothers and 74% of the household heads being illiterate.

**Table 1:** Characteristics of the study population (*N* = 1,439).

**Variables**		***N***	**%**
Strata	Cissé	251	17
	Kodougou	175	12
	Nouna	208	14
	Sono	143	10
	Toni	662	46
Child's age^*^	In months	1,439	37 (25)
Child's sex	Girls	748	52
Siblings aged <5 years	Yes	1,119	78
Fever episodes over the past 2 weeks	Yes	449	32
Diarrhea over the past 2 weeks	Yes	231	16
Currently breastfeeding	Yes	368	26
HAZ^*^	*Z*-score	1,439	−1.2 (1.49)
WHZ^*^	*Z*-score	1,434	−0.37 (1.25)
Stunting	Yes	345	24
Wasting	Yes	85	6
Wealth quint.	Poorest	282	20
	Poor	283	20
	Average	279	20
	Rich	308	22
	Richest	254	18
Mother's ethnicity	Dafing	452	31
	Bwaba	317	22
	Mossi	302	21
	Peul	263	18
	Other	102	7
Household head's ethnicity	Dafing	445	31
	Bwaba	322	22
	Mossi	280	19
	Peul	287	20
	Other	105	7
Mother's education	Illiterate	1,125	79
	Literate	70	5
	Primary	186	13
	Secondary	49	3
Household head's education	Illiterate	1,058	74
	Literate	159	11
	Primary	181	13
	Secondary	37	3

* *Median (IQR)*.

Supplementary Table S1 displays the time trends of the 15 rainfall indicators by the five strata for the years 1981–2019. According to the data, the Nouna HDSS area has one rainy season from May to October. The total annual rainfall (PRCPTOT) was the lowest in Cissé with a mean of 724 mm and the highest in Kodougou with a mean of 778 mm combined from 1981 to 2019. The time trend was statistically significant and positive across all five strata. Over 30% of the annual rain falls in August and another 25% falls in July (PRCPJUL). Both months together cover two third of the very heavy precipitation days (*R* ≥ 20 mm). So-called mini-droughts or consecutive dry days during the wet season (CDDws) are common in the region and take on average 10 days. Statistically significant differences between strata (spatial variability) were detected for three rainfall indicators: The number of consecutive wet days (CWD), for very wet (R95p) and for extremely wet days (R99p).

As the absolute numbers were transferred to z-scores to provide a common metric unit for further analysis, Supplementary Table S2 presents the means and z-scores of the rainfall indicators combined for the five strata. *Z*-scores of the rainfall indicators ranged between −0.33 and 0.90. Negative (positive) *z*-scores indicate a negative (positive) deviation from the mean reference (1981 to 2019) and so a reduction (or increase, respectively) in their occurrence.

### Dietary Patterns and Their Associations With Child Nutritional Status

We sought to explore the hypotheses that (i) rainfall variability is associated with the composition of the children's diet, and that (ii) rainfall variability related to the children's diet is associated with their nutritional status. The association between dietary patterns and child HAZ and WHZ in the Nouna HDSS was previously established for the year 2018 (20). This analysis was replicated for the years 2017 to 2019, and the results are shown in Supplementary Table S3.

By PCA, three distinct dietary patterns were identified that explained 26% of the total variation in food intake by children 7–60 months of age in the Nouna HDSS area (Table 2). The dietary patterns were labeled based on their positive correlations with food item intakes as “market-based” (DP1), “legume-based” (DP2), and “vegetable-based” (DP3) and explained 10, 8, and 8%, respectively, of the variation in food groups intake in the study population. DP1 was characterized by pasta, eggs, poultry, sweets, bread, beverages, rice and cassava (market-based), DP2 was characterized by soumbala, oils and fats, dark green leaves, peanuts, millet, tea and sorghum, and DP3 was characterized by oils and fats, okra, tomatoes, eggplant, maize, coffee, and fish.

**Table 2:** Rotated factor loadings of food items for the three dietary patterns among 1,439 children aged <5 years in the Nouna HDSS area.

**Food groups**	**DP1**	**DP2**	**DP3**
	**Market-based**	**Legume-based**	**Vegetable-based**
	**diet**	**diet**	**diet**
Pasta	0.57^*^	0.24	0.10
Eggs	0.56^*^	−0.07	0.05
Poultry	0.55^*^	−0.03	0.09
Sweets	0.52^*^	0.19	0.25
Bread	0.49^*^	0.07	0.06
Beverages	0.46^*^	−0.11	0.01
Rice	0.45^*^	0.40	0.03
Cassava	0.41^*^	0.06	−0.09
Soumbala	0.05	0.60^*^	0.09
Oils and fats	−0.01	0.57^*^	0.42^*^
Dark green leaves	0.26	0.46^*^	0.03
Peanuts	0.35	0.41^*^	−0.06
Millet	−0.09	0.41^*^	−0.03
Tea	0.10	0.41^*^	0.02
Okra	−0.05	0.05	0.70^*^
Tomatoes	0.08	−0.02	0.66^*^
Eggplant	0.07	0.14	0.64^*^
Maize	0.09	−0.17	0.46^*^
Coffee	0.21	0.04	0.43^*^
Fish	0.16	0.29	0.42^*^
Meat	0.38	0.37	−0.03
Cabbage	0.37	0.13	−0.08
Cowpea beans	0.27	0.02	0.28
Animal milk	0.26	0.30	0.12
Onions	0.25	0.39	−0.09
Fruits	0.25	0.11	0.19
Couscous	0.20	0.24	0.02
Groundnuts	0.18	0.13	−0.13
Mother's milk	0.02	−0.36	−0.03
Sorghum	−0.08	0.27	0.01
**Explained variance**	**9.88%**	**8.28%**	**7.87%**

* *Food groups with factor loadings of ≥|0.40| indicate relevant contributions to the DPS*.

The market-based and the legume-based dietary patterns were positively and significantly associated with HAZ in the fully adjusted model. For each score-point increase of the market-based dietary pattern, HAZ was higher by 0.02 (95% CI: 0.00, 0.03, *p* < 0.05); and for each score-point increase of the legume-based dietary pattern, HAZ was higher by 0.01 (95% CI: 0.00, 0.02, *p* < 0.05). For WHZ, none of the fully adjusted models showed a statistically significant association with the three dietary patterns. Only the unadjusted model for the vegetable-based diet was statistically significantly associated with WHZ, showing a 0.01 higher WHZ per 1 score-point increase (95% CI: 0.00, 0.02, *p* < 0.05).

### The PVS and Its Association With Dietary Patterns

**Figure 3:**
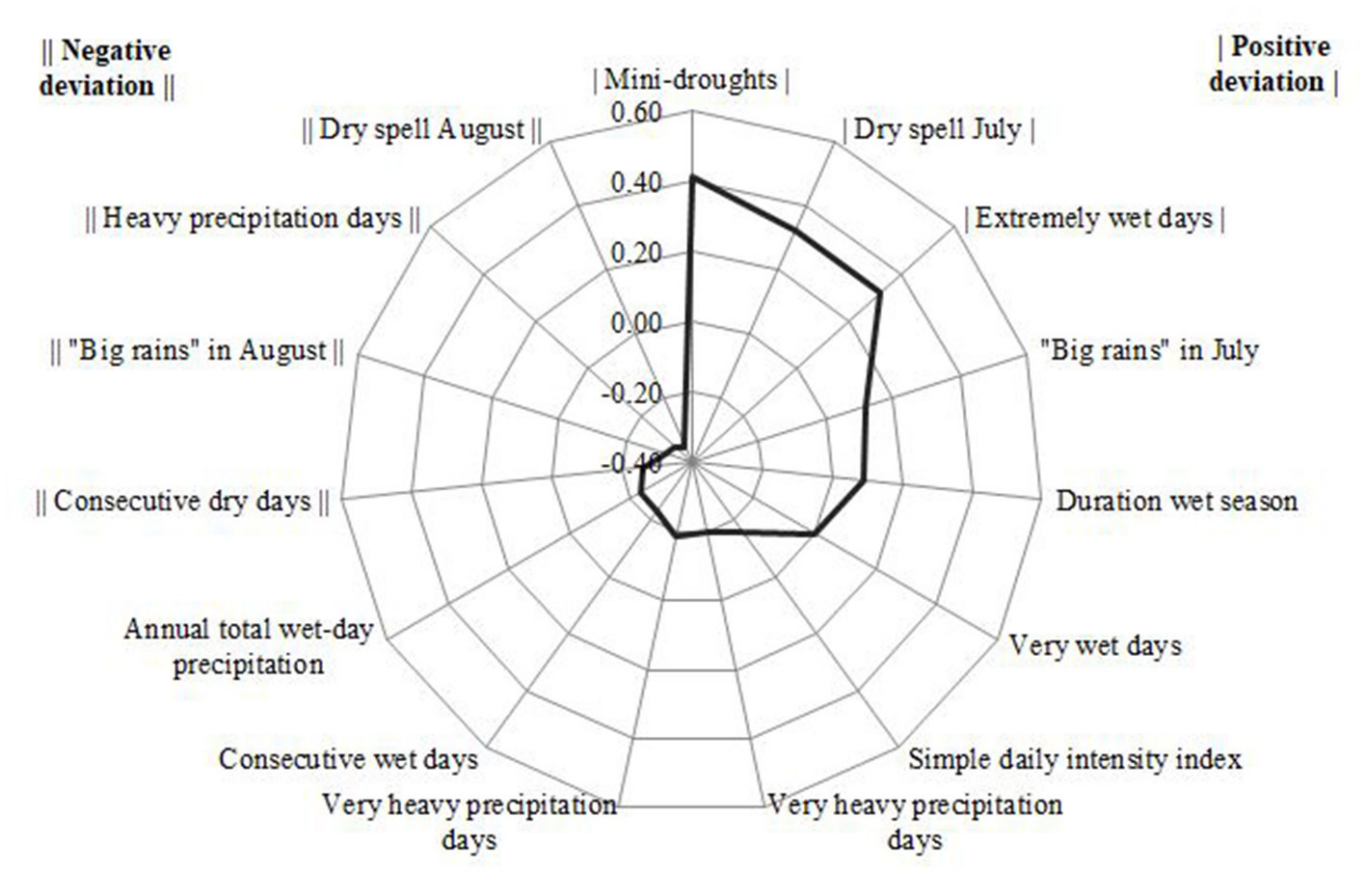
Visualization of the RRR rotated factor loadings of the rainfall indicators that best describe the RRR-derived PVS. Rainfall indicators with factor loadings of ≥|0.20| indicate relevant contributions to the PVS. A positive deviation indicates a higher manifestation (increase) of the respective rainfall indicators, while a negative deviation indicates a lower manifestation (reduction).

The characteristics of the RRR-derived PVS are presented in Figure 3 and Supplementary Table S4. Based on the RRR output, the PVS explained 17% of the variance in rainfall indicators (the predictor variables) and 14% of the total variance in DPS (the response variables). The PVS can, thus, be considered predictive for diets with a higher PVS indicating a higher adherence to and so consumption of all dietary patterns by the children in our sample. An increase in the PVS translates into an increased deviation from the norm (the PVS ranges from −3.85 to 2.85 SDs). The strongest positive relationship was seen with the vegetable-based dietary pattern, followed by the market-based and the legume-based dietary pattern. In order to underline the strength of the association between the rainfall indicators and the three DPS, the Pearson correlation coefficients of the PVS are presented in Supplementary Table S5. Here, the partial correlation coefficients for the precipitation indicators and the three DPS ranged from −0.61 to 0.71 (adjusted for age and sex of the child and stratum). The strongest correlations of the rainfall indicators were seen with the vegetable-based dietary pattern. It confirms that an increasing PVS supports a higher consumption of the vegetable-based, market-based and legume-based diet.

The PVS itself was characterized based on the factor loadings that showed ≥|0.20| (Figure 3). Factor loadings above this number indicate a relevant contribution to the variability score, while a positive factor loading indicates an upward deviation (increase) of the respective rainfall indicator and a negative factor loading indicates a downward deviation (reduction).

### The Associations of the PVS With Children's Nutritional Status

Table 3 displays the associations of the PVS with the continuous outcome variables HAZ and WHZ, respectively. There was an inverse association of the PVS with HAZ and a positive association of the PVS with WHZ. Both showed statistically significant results. A 1-point increase in the PVS was associated with a reduction in HAZ by 0.029 (95% CI: −0.06, −0.00, *p* < 0.05) in the unadjusted model. The associations with HAZ did not change after adjustments for covariates as shown in model 1 and 2. Thus, a 1 SD increase of the PVS translated into an increase in stunting prevalence to 24.6%. In the case of WHZ, a 1-point score increase in the PVS was positively associated with WHZ, which increased by 0.013 (95% CI: −0.01, 0.04, *p* > 0.05) in the unadjusted model. A statistically significant association only occurred in the adjusted models. WHZ improved by 0.032 (95% CI: 0.01, 0.06, *p* < 0.05) in the fully adjusted model and so indicated a mediating effect through socio-economic factors. Here, a 1 SD increase of the PVS translated into a decrease in wasting prevalence to 5.5%.

**Table 3:** Multilevel regression models (child-household-village) examining the factors of the PVS on HAZ and WHZ of young children in the Nouna HDSS area.

**Adherence to the PVS**	**Ref. tertile**	**Second tertile**		**Third tertile**		**Per 1-point increase of the**	
	**Low**	**Medium**		**High**		**PVS**	
**HAZ (** ***N*** **=** **1,364)**	**ß-coef**.	**ß-coef**.	**95% CI**	**ß-coef**.	**95% CI**	**ß-coef**.	**95% CI**
Unadj. model	0.00	−0.008	−0.02, 0.01	−0.012	−0.03, −0.01	−0.029^*^	−0.06, −0.00
Adj. model 1^a^	0.00	−0.004	−0.02, 0.01	−0.010	−0.02, 0.03	−0.028	−0.06, −0.00
Adj. model 2^b^	0.00	−0.003	−0.02, 0.01	−0.010	−0.02, 0.04	−0.028	−0.06, 0.00
**WHZ (** ***N*** **=** **1,359)**	**ß-coef**.	**ß-coef**.	**95% CI**	**ß-coef**.	**95% CI**	**ß-coef**.	**95% CI**
Unadj. model	0.00	−0.009	−0.02, 0.00	0.003	−0.01, 0.01	0.013	−0.01, 0.04
Adj. model 1^a^	0.00	−0.008	−0.02, 0.00	0.008	−0.00, 0.02	0.027^*^	0.00, 0.05
Adj. model 2^b^	0.00	−0.005	−0.02, 0.01	0.011	−0.00, 0.02	0.032^*^	0.01, 0.06

a *Adj. for child's age and sex*;

b *Adj. for all variables included in adj. model 1 and mother's and household head's education and ethnicity, household wealth, siblings aged <5 years, child's fever and diarrhea the previous two weeks, and breastfeeding status*;

* *p < 0.05*.

In order to investigate the sensitivity of the PVS to the nutritional status, we also investigated the association with a higher rainfall variability defined by an increase in the PVS by 2 and 3 SD. With a HAZ reduction by −0.06 and −0.09, this translated into a stunting prevalence of 25.2 and 25.8%, respectively. With a WHZ increase by 0.06 and 0.09, this translated into wasting prevalence of 5 and 4.5%, respectively.

## Discussion

### Rainfall Variability in Burkina Faso

This study provides an empirical approach on how rainfall variability can be linked to dietary patterns and child undernutrition. Only a few studies have described rainfall trends over time specifically in Burkina Faso given the sparse number of weather stations and a lack of high-quality, long-term data (1, 2, 47, 67, 68). According to climate data from 1950 to 2013 for Burkina Faso from De Longueville et al. (12), an overall decline of total annual rainfall can be observed since the 1950s with a slight recovery of rainfall since the late 1990s following the severe droughts of the 1970s and 1980s (69). Equally, there is a decrease in the number of rainy days (69), a downward trend in the number of consecutive dry days during the wet season (mini-droughts), as well as a decrease in the length of the wet season in Burkina Faso (12). Our data confirms this observed precipitation variability, while keeping in mind inter-annual and spatial variability (7, 13, 69). We can confirm an increase in cumulative annual rainfall over a shorter period of time and thus, an increase in extreme rainfall events such as in the number of (very) heavy precipitation days. An upward trend in heavy rainfall was found specifically in July and August with more than 60% of the total annual rainfall (over 400 mm) coming down. This aligns with the observations made by Hondula et al. (2) in the same study region. These 2 months define the success or respective failure of the harvest output of subsistence farmers and thus, whether they will be able to have enough food until the next harvest to feed their families and provide financial income (22, 61). Overall, a well-distributed and quantitatively sufficient rainfall is essential for crop growth (18).

### The Impact of Rainfall Variability on Child Nutritional Status

In order to measure the impact of the recognized rainfall variability, we applied RRR as a dimension-reduction technique. Although RRR originates from nutrition epidemiology and is a novel approach in this interdisciplinary context, we identified it as an alternative empirical approach to display rainfall variability than using a single indicator, like it is mostly done in the identified literature. A single indicator oversimplifies the complexity and interlinkage of climate events (65, 70). So far, studies that explored the link between climate and child nutritional status often referred to cumulative annual rainfall or temperature shocks (as standard deviations of the norm) (23, 71, 72), combined various environmental or topographic indicators (e.g., NDVI, altitude, relief of terrain or living location) (21, 73, 74) or investigated seasonal differences (e.g., years of drought, monsoon season, born during the rainy season, birth month) (38, 75, 76). None applied a climate or rainfall variability pattern as proposed here. Additionally, we only found a single publication that explicitly addressed the link between climate variability and diets, but excluded child undernutrition (77). Here, the authors associated rainfall variability in the previous year to child dietary diversity in a multi-country analysis. For West Africa, an increase in precipitation was found to increase dietary diversity independent of mediating socio-economic factors. Overall, diets or dietary patterns, respectively, have not yet been considered as a mediator between climate and nutritional status (78, 79); or have been missed to integrate weather data, when associations between child undernutrition and seasonality of diets were discussed (64, 80).

An advantage of the RRR-approach was that it allowed us to define the association of the PVS with diets and nutritional status for the year before the nutrition assessment. It has not yet been defined which climate timing predicts and explains child nutritional outcomes the best. Accordingly, we identified studies that used sum of rainfall (and temperature) by trimester prior to the date of birth for associations with birth weight outcomes (37, 38). Here, the authors assumed that child undernutrition manifests itself already *in utero* or even before conception due to heat stress and dehydration of the mother. With regard to child stunting and wasting, two time points were chosen by Skoufias and Vinha (72) to link weather shocks with chronic undernutrition of children in Mexico. They found that positive rainfall shocks (=more severe rainfall than normal) in the preceding agricultural year showed to have a negative impact on average HAZ (−0.87 and −0.32 coefficient points, respectively). This finding can also be confirmed for our PVS and HAZ, which changed by −0.029 with an increase in the PVS. Cooper et al. (78) examined the association of extreme rainfall and droughts on child anthropometry for even five different time periods. They found that short-term rainfall shocks over 12 months were significant predictors of WHZ, while periods of 36 months were significantly associated with HAZ in Ghana and Bangladesh. Equally, increased annual rainfall worsened the nutritional outcomes in both cases.

### Mediating Effects of Socio-Economic Factors to Weather Impacts

Our findings additionally showed that socio-economic factors may play a role in mediating the associations of rainfall variability and child undernutrition. A mediating effect was specifically found for WHZ (0.19 change), but less directional for HAZ (0.01 change). In general, a risk for wasting is higher due to infectious diseases, which appreciate a wetter environment for breading and transmission due to lack of hygiene (81). Shively (82) found that greater road density and improved access to health facilities mitigate a child's risks for undernutrition to variations in precipitation in Nepal and Uganda; to name just one. Already small positive social and environmental developments are assumed to have positive mitigating effects on child growth (37, 83, 84).

In this study, we also hypothesized a mitigating effect of diets on child nutritional outcomes depending on rainfall variability. Accordingly, the PVS explained most of the DPS with vegetables (25%) and with market-related products (11%). Those were characterized by frequent intakes of maize and rice, respectively. Both food items are common in this subsistence farming environment and also less drought tolerant than sorghum and millet (1, 85). This might explain that the legume-based dietary pattern, which was characterized by sorghum and millet, changed less (8%) in relation to the PVS. Since we conducted the study during the lean season, when stocks tend to be empty from the previous year's harvest, those diets may indicate an adaptation response to the weather. Subsequently, although subsistence farmers strongly rely on those food crops for a living, they may have adapted their diets by either reducing the number of meals per day, acquiring imported foods from the market, if the financial situation allows, or referring to vegetable gardens maintained during the dry season (22, 86, 87).

### Study Limitations and Prospects for Further Research

We would like to encourage further research in the application of RRR in other settings to investigate its feasibility in interdisciplinary research on climate change, nutrition and public health. Given our data-driven approach applying two dimension-reduction techniques, the analyses are easily reproducible in other geographic locations using available data. The statistical application is well-known and documented. Our aim was to explore an unconventional approach originating from nutrition epidemiology and investigating its applicability in a complex context such as climate, nutrition and health. Yet, our results might be a reflection of the indirectness of the methods used, which needs to be further explored.

Additionally, we encourage the inclusion of temperature indicators (70). Although temperatures tend to be lower during the rainy season due to increased cloud cover, which suppresses incoming solar radiation, and increased cooling due to evaporation of ground and atmosphere moisture (2), some studies have shown that temperature had a direct effect on child health outcomes (38) and increased risk of mortality among children aged <5 years (68, 88). Equally the temporal longitudinal scales from conception to early childhood and any carry on/over effects of climate impacting children's nutritional status should be further investigated. A child starts to be exposed to environmental impacts already through the mother even before conception, and continues through pregnancy, childhood and adulthood (29). Subsequently, the diet and living conditions of a mother prior to a child's birth may have (additional) implications on health and development (37). Equally, it may require a longer time window to investigate the lagged health effect of diet on the nutritional status of the child than was possible in the present study. In this regard we recognize the limitations of our study (i) that we have only data for one time point (the lean and rainy season) and (ii) that we only collected data during one season. Those aspects may have added potential bias to our results. Although the diets were found similar within the season (internal validity), they are likely to differ between seasons (external validity) (64). To further consider for various temporal scales than applied in this study, long-term climate and nutrition data would be needed.

### Implications for Policy Action

Evidence-based findings on the development of the current climate as well as long-term projections on future climate change and its impact on nutrition and public health provide a first step to promote policy action and so the development of long- and short-term adaptation strategies. Adaptation to climate change has become a requirement to assure sustainability of agriculture production, food security, health, and nutrition and improve resilience of the respective population (89). Yet, given the multi-sectoral determinants of child undernutrition, there is not a single intervention that can act as a “silver bullet” to reduce and/or prevent a rise (90). Climate change and so climate extremes pose an increased risk to children, who are in general more vulnerable to infectious diseases, but also face a vicious cycle once already undernourished (91). Subsequently, political actions should integrate climate predictions and weather forecasts in their decision making to act and prevent the occurrence of child health risks. Adaptation interventions such as on food diversification or home gardening can mitigate the negative impacts of climate change in the long- and short-run (87, 92, 93). Yet, poor and vulnerable population groups rely on the governments to promote and subsidize them.

## Conclusion

West Africa experienced increasing rainfall variability in the past decades as a component of ongoing climate change. This includes an increase in total annual rainfall, more extreme rainfall events, regular mini-droughts during the rainy season, and a shorter rainy season overall; which was also shown for a small area in Burkina Faso. The Nouna HDSS area was specifically of interest due to its continued high prevalence of stunting (24%) and wasting (6%) of children aged <5 years. We explored (i) the association of rainfall variability with the children's diets by constructing a precipitation variability score (PVS), and (ii) established its association with child undernutrition. Our results showed that an increase in rainfall variability has a stronger impact on chronic than acute undernutrition considering the mediating effect of socio-economic factors. This should be kept in mind when setting-up and evaluating adaptation measures. Equally, social and environmental investments should be further promoted to counteract climate change impacts.

## Data Availability Statement

The raw data supporting the conclusions of this article will be made available by the authors, without undue reservation. The data will be made available by the corresponding author upon reasonable request.

## Ethics Statement

The studies involving human participants were reviewed and approved by Heidelberg University Ethical Committee, Medical Faculty, Alte Glockengießerei 11/1, 69115 Heidelberg, Germany. Written informed consent to participate in this study was provided by the participants' legal guardian/next of kin. The study was conducted in accordance with the most recent version of the ethical principles of the World Medical Association's Declaration of Helsinki, which is applicable for national and international regulatory requirements. Ethical approval was obtained both from the Heidelberg University Ethical Committee (S-180/2017), Germany, and the Nouna Health Research Center Ethical Committee, Burkina Faso. All caregivers participating in the study provided informed written consent. Parents of severely undernourished children were encouraged to visit the closest health care center for consultation. We provided a referral document with financial support for the visit.

## Author Contributions

IM was responsible for the data acquisition, statistical analysis, data interpretation, and manuscript drafting. KB provided statistical guidance on the analyses and their interpretation. JB derived the rainfall dataset and contributed to the understanding of the rainfall measurements. IT contributed to the data acquisition and interpretation. PW reviewed data interpretation and paper logic. RS provided the conceptual framework, the study design, and guided the data acquisition. ID contributed to the methodology, statistical analyses, and interpretation of the data. All authors contributed to the writing of the paper, critically reviewed it, and gave final approval for its publication.

## Funding

The lead author was financially supported by the Robert Bosch Foundation, through a PhD fellowship provided by the Baden-Württemberg Landesgraduiertenförderung Program (LGF) from 2017 to 2020 and through a DAAD stipend provided in 2017. The data collection was funded by the Heidelberg Institute of Global Health (HIGH) from 2017 to 2019. This work is part of the research unit Climate Change and Health in sub-Saharan Africa, which received funding from the German Research Foundation (DFG) (reference: DA 1881/3-1).

## Conflict of Interest

The authors declare that the research was conducted in the absence of any commercial or financial relationships that could be construed as a potential conflict of interest.

## Publisher's Note

All claims expressed in this article are solely those of the authors and do not necessarily represent those of their affiliated organizations, or those of the publisher, the editors and the reviewers. Any product that may be evaluated in this article, or claim that may be made by its manufacturer, is not guaranteed or endorsed by the publisher.
