# Supplementary material for: The Impact of Rainfall Variability on Diets and Undernutrition of Young Children in Rural Burkina Faso
Source: Front Public Health. 2021 Sep 20;9:693281. doi: 10.3389/fpubh.2021.693281 (PMC8489680; doi:10.3389/fpubh.2021.693281)
Supplement: Supplementary file 1 [file Data_Sheet_1.pdf]

*Supplementary Material*

## Supplementary Table S1: 15 rainfall indicators by stratum in the Nouna HDSS area from 1981 to 2019

| Indicator | | | Cissé | | | Kodougou | | | Nouna | | | Sono | | | Toni | | | Diff. in means |
|---|---|---|---|---|---|---|---|---|---|---|---|---|---|---|---|---|---|---|
| **ID (unit)** | **Indicator name** | **Definitions** | **Mean ± SD** | **Min-Max** | **Trend[a]** | **Mean ± SD** | **Min-Max** | **Trend[a]** | **Mean ± SD** | **Min-Max** | **Trend[a]** | **Mean ± SD** | **Min-Max** | **Trend[a]** | **Mean ± SD** | **Min-Max** | **Trend[a]** | **p-value** |
| PRCPTOT (mm) | Annual total precipitation | Annual total PRCP in wet days (RR>=1mm) | 724 ± 166 | 355 - 1087 | ↑* | 778 ± 153 | 430 - 1113 | ↑** | 730 ± 127 | 540 - 1019 | ↑* | 730 ± 135 | 486 - 1062 | ↑* | 744 ± 139 | 504 - 1115 | ↑** | 0.477 |
| R95p (days) | Very wet days | Annual number of days with RR>95th percentile | 5 ± 1 | 0 - 6 | ↑* | 4 ± 2 | 0 - 6 | ↗ | 5 ± 1 | 0 - 6 | ↑ | 4 ± 2 | 0 - 6 | ↑ | 4 ± 2 | 0 - 6 | ↑ | 0.1091 |
| CDD (days) | Consecutive dry days | Max. no. of consecutive days with RR<1mm | 171 ± 34 | 64 - 222 | ↑* | 164 ± 37 | 81 - 238 | ↗ | 161 ± 33 | 97 - 238 | ↑* | 164 ± 31 | 80 - 236 | ↗ | 161 ± 34 | 63 - 211 | ↗ | 0.715 |
| R99p (days) | Extremely wet days | Annual number of days with RR>99th percentile | 2 ± 2 | 0 - 6 | ↑ | 1 ± 2 | 0 - 5 | → | 1 ± 2 | 0 - 5 | ↗ | 4 ± 2 | 0 - 6 | ↑ | 2 ± 2 | 0 - 5 | ↗ | **0.000**** |
| R20Aug (days) | Days with very heavy rains in Aug | Count of days when PRCP>=20mm | 5 ± 2 | 0 - 9 | ↗* | 5 ± 2 | 0 - 9 | ↗* | 4 ± 2 | 1 - 8 | ↗** | 5 ± 2 | 0 - 9 | ↗ | 5 ± 2 | 2 - 8 | ↗* | 0.787 |
| PRCPAUG (mm) | Total precipitation in Aug | Cumulative rainfall in August (RR>=1mm) | 243 ± 106 | 57 - 561 | ↗* | 227 ± 68 | 150 - 480 | ↗** | 224 ± 76 | 87 - 379 | ↗** | 231 ± 82 | 78 - 378 | ↗ | 230 ± 75 | 124 - 397 | ↗** | 0.869 |
| PRCPJUL (mm) | Total precipitation in July | Cumulative rainfall in July (RR>=1mm) | 181 ± 66 | 63 - 300 | ↗* | 196 ± 63 | 85 - 359 | → | 183 ± 51 | 64 - 298 | → | 182 ± 59 | 70 - 367 | → | 193 ± 67 | 32 - 316 | ↗** | 0.737 |
| R20Jul (days) | Days with very heavy rains in July | Count of days when PRCP>=20mm | 4 ± 2 | 1 - 8 | ↗ | 4 ± 2 | 1 - 8 | → | 4 ± 2 | 1 - 7 | → | 3 ± 2 | 1 - 9 | → | 4 ± 2 | 0 - 8 | ↗* | 0.793 |
| R10 (days) | Days with heavy precipitation | Annual count of days when PRCP>=10mm | 25 ± 5 | 15 - 37 | ↗* | 27 ± 5 | 18 - 39 | ↗** | 25 ± 4 | 17 - 33 | ↗ | 25 ± 4 | 19 - 32 | →** | 25 ± 4 | 18 - 38 | →* | 0.360 |
| SDII (mm/day) | Simple daily intensity index | Annual total precipitation by no. of wet days (PRCP>=1mm) | 15 ± 3 | 9 - 22 | ↗** | 15 ± 2 | 12 - 20 | → | 15 ± 2 | 11 - 20 | → | 15 ± 2 | 9 - 20 | → | 15 ± 2 | 12 - 19 | →* | 0.717 |
| R20 (days) | Days with very heavy precipitation | Annual count of days when PRCP>=20mm | 13 ± 4 | 3 - 21 | ↗ | 14 ± 3 | 6 - 21 | →* | 13 ± 3 | 7 - 20 | → | 13 ± 4 | 4 - 23 | → | 13 ± 3 | 8 - 19 | →* | 0.437 |
| R25 (days) | Days with very heavy precipitation | Annual count of days when PRCP>=25mm | 9 ± 4 | 1 - 17 | ↗* | 10 ± 3 | 3 - 17 | → | 9 ± 3 | 3 - 15 | → | 9 ± 3 | 1 - 16 | → | 10 ± 3 | 5 - 18 | →** | 0.325 |
| CWD (days) | Consecutive wet days | Max. no. of consecutive days with RR>=1mm | 4 ± 1 | 2 - 9 | → | 4 ± 2 | 3 - 8 | → | 3 ± 1 | 2 - 6 | → | 4 ± 1 | 3 - 6 | → | 4 ± 1 | 2 - 8 | → | **0.015*** |
| Lws (days) | Duration wet season | Length of the wet season | 132 ± 23 | 88 - 177 | → | 142 ± 21 | 94 - 179 | → | 136 ± 23 | 77 - 179 | → | 138 ± 24 | 85 - 182 | → | 135 ± 23 | 77 - 177 | → | 0.359 |
| CDDws (days) | Consecutive dry days in wet season (mini-drought) | Max. no. of consecutive dry days (RR<1 mm) during wet season | 10 ± 3 | 3 - 17 | → | 11 ± 2 | 7 - 15 | → | 9 ± 3 | 4 - 15 | ↘ | 11 ± 3 | 5 - 17 | → | 10 ± 3 | 5 - 17 | ↘* | 0.182 |

[a] Slope = steep increase (↑), when > 1.0; light increase (↗), when < 1.00 and > 0.10; no change (→), when > -0.10 and < 0.10; light decrease (↘), when < -0.10; * p-value < 0.05, ** p-value < 0.01

## Supplementary table S2: Means (SDs) and z-scores of the rainfall indicators

| ID (unit) | Indicator name | Mean (SD) | Z-score |
|---|---|---|---|
| PRCPTOT (mm) | Annual total precipitation | 852 (150) | 0.79 |
| SDII (mm/day) | Simple daily intensity index | 17 (2) | 0.66 |
| R10 (days) | Days with heavy precipitation | 28 (5) | 0.70 |
| R20 (days) | Days with very heavy precipitation | 15 (3) | 0.48 |
| R25 (days) | Days with very heavy precipitation | 12 (3) | 0.68 |
| CDD (days) | Consecutive dry days | 162 (41) | -0.02 |
| CWD (days) | Consecutive wet days | 4 (1) | 0.16 |
| R95p (mm) | Very wet days | 5 (1) | 0.43 |
| R99p (mm) | Extremely wet days | 3 (2) | 0.52 |
| Lws (days) | Duration wet season | 128 (14) | -0.33 |
| CDDws (days) | Consecutive dry days in wet season (mini-drought) | 10 (3) | -0.14 |
| R20Jul (days) | Days with "big rains" in July | 4 (1) | 0.31 |
| R20Aug (days) | Days with "big rains" in August | 6 (2) | 0.61 |
| PRCPJUL (mm) | Total precipitation in July | 227 (49) | 0.59 |
| PRCPAUG (mm) | Total precipitation in August | 300 (82) | 0.90 |

**Supplementary table S3: Associations of the three DPSs with HAZ of 1,439 children and WHZ of 1,434 children aged 7-60 months**

| | **Tertile 1** | **Tertile 2** | | **Tertile 3** | | **Per 1 score-point increase** | | |
|---|---|---|---|---|---|---|---|---|
| | | ß-coef. | 95% CI | ß-coef. | 95% CI | ß-coef. | 95% CI | p-value trend |
| ***Height-for-Age zscore (HAZ)*** | | | | | | | | |
| **DPS1: Market-based diet** | | | | | | | | |
| Unadjusted model | Ref. | -0.02 | -0.20, 0.15 | 0.07 | -0.10, 0.24 | 0.01 | 0.00, 0.02 | 0.222 |
| Adj. model 1a | Ref. | 0.07 | -0.11, 0.23 | 0.19 | 0.02, 0.36 | 0.02 | 0.00, 0.03 | **0.010*** |
| Adj. model 2b | Ref. | 0.03 | -0.13, 0.20 | 0.19 | 0.02, 0.35 | 0.02 | 0.00, 0.03 | **0.012*** |
| **DPS2: Legume-based diet** | | | | | | | | |
| Unadjusted model | Ref. | -0.02 | -0.19, 0.16 | -0.13 | -0.29, 0.03 | -0.01 | -0.02, 0.00 | 0.115 |
| Adj. model 1a | Ref. | 0.12 | -0.05, 0.30 | 0.06 | -0.10, 0.23 | 0.00 | -0.01, 0.01 | 0.383 |
| Adj. model 2b | Ref. | 0.19 | 0.01, 0.37 | 0.17 | 0.00, 0.34 | 0.01 | 0.00, 0.02 | **0.045*** |
| **DPS3: Vegetable-based diet** | | | | | | | | |
| Unadjusted model | Ref. | -0.03 | -0.20, 0.14 | 0.04 | -0.14, 0.21 | 0.00 | -0.01, 0.01 | 0.991 |
| Adj. model 1a | Ref. | 0.03 | -0.14, 0.20 | 0.09 | -0.08, 0.26 | 0.01 | -0.01, 0.02 | 0.353 |
| Adj. model 2b | Ref. | -0.07 | -0.23, 0.10 | 0.00 | -0.18, 0.17 | 0.00 | -0.01, 0.01 | 0.818 |
| ***Weight-for-Height zscore (WHZ)*** | | | | | | | | |
| **DPS1: Market-based diet** | | | | | | | | |
| Unadjusted model | Ref. | 0.03 | -0.10, 0.16 | 0.00 | -0.13, 0.13 | 0.00 | -0.01, 0.01 | 0.444 |
| Adj. model 1a | Ref. | -0.02 | -0.15, 0.11 | -0.06 | -0.19, 0.07 | 0.00 | -0.01, 0.01 | 0.995 |
| Adj. model 2b | Ref. | -0.04 | -0.17, 0.09 | -0.09 | -0.23, 0.04 | 0.00 | -0.01, 0.01 | 0.573 |
| **DPS2: Legume-based diet** | | | | | | | | |
| Unadjusted model | Ref. | 0.06 | -0.07, 0.19 | 0.05 | -0.08, 0.18 | 0.01 | 0.00, 0.01 | 0.084 |
| Adj. model 1a | Ref. | -0.04 | -0.17, 0.09 | -0.07 | -0.20, 0.07 | 0.00 | -0.01, 0.01 | 0.754 |
| Adj. model 2b | Ref. | -0.04 | -0.18, 0.10 | -0.04 | -0.19, 0.10 | 0.00 | -0.01, 0.01 | 0.936 |
| **DPS3: Vegetable-based diet** | | | | | | | | |
| Unadjusted model | Ref. | 0.06 | -0.07, 0.19 | 0.08 | -0.05, 0.22 | 0.01 | 0.00, 0.02 | **0.047*** |
| Adj. model 1a | Ref. | -0.01 | -0.14, 0.12 | 0.05 | -0.09, 0.18 | 0.01 | 0.00, 0.02 | 0.166 |
| Adj. model 2b | Ref. | -0.04 | -0.17, 0.09 | 0.03 | -0.11, 0.16 | 0.00 | -0.01, 0.01 | 0.435 |

a Adjusted for child's age and sex, and stratum; b Adjusted for all variables included in adj. model 1 and mother's and household head's education and ethnicity, household wealth, siblings aged <5 years, child's fever and diarrhea the previous two weeks, and breastfeeding status, and year of data collection; * p-value < 0.05

**Supplementary table S4: RRR-derived explained variation and rotated factor loadings of rainfall indicators with the three DPSs**

| **Extracted factors** | | | **Explained variation (%)** | **Factor loadings** | **Factor weights** |
|---|---|---|---|---|---|
| ***Predictor variables*** | | | | | |
| CDDws | Mini-droughts | Max. number of consecutive dry days (RR<1 mm) during wet season | 49.06 | **0.41** | 0.80 |
| PRCPJUL | Dry spell July | Monthly total PRCP in wet days (RR>=1mm) | 29.96 | **0.32** | 0.81 |
| R99p | Extremely wet days | Annual total PRCP when RR>99th percentile | 30.42 | **0.32** | -0.27 |
| R20Jul | Number of "big rains" in July | Count of days when PRCP>=20mm | 4.57 | 0.12 | -0.49 |
| Lws | Duration wet season | Length of the wet season | 2.59 | 0.09 | -0.34 |
| R95p | Very wet days | Annual total PRCP when RR>95th percentile | 0.00 | 0.00 | -0.27 |
| SDII | Simple daily intensity index | Annual total precipitation by number of wet days (PRCP>=10mm) | 7.89 | -0.16 | -0.41 |
| R20 | Number of very heavy precipitation days | Annual count of days when PRCP>=20mm | 11.64 | -0.20 | 0.18 |
| R25 | Number of very heavy precipitation days | Annual count of days when PRCP>=25mm | 11.23 | -0.19 | 0.84 |
| CWD | Consecutive wet days | Maximum number of consecutive days with RR>=1mm | 15.24 | -0.23 | 0.07 |
| PRCPTOT | Annual total wet-day precipitation | Annual total PRCP in wet days (RR>=1mm) | 15.66 | -0.23 | -0.41 |
| CDD | Consecutive dry days | Maximum number of consecutive days with RR<1mm | 19.77 | **-0.26** | -0.31 |
| R20Aug | Number of "big rains" in August | Count of days when PRCP>=20mm | 29.48 | **-0.32** | 0.14 |
| R10 | Number of heavy precipitation days | Annual count of days when PRCP>=10mm | 32.63 | **-0.33** | 0.11 |
| PRCPAUG | Dry spell August | Monthly total PRCP in wet days (RR>=1mm) | 36.48 | **-0.35** | 0.00 |
| **Explained variance (%)** | | | **19.77** | | |
| ***Response variables*** | | | | | |
| DP 1 | Market-based diet | | 10.11 | | 0.49 |
| DP 2 | Legume-based diet | | 7.71 | | 0.41 |
| DP 3 | Vegetable-based diet | | 24.51 | | 0.77 |
| **Explained variance (%)** | | | **14.11** | | |

* Precipitation indicators with factor loadings of ≥ |0.20| indicate relevant contributions to the precipitation pattern score

**Supplementary table S5: Pearson correlation coefficients (N=1,364) for precipitation indicators (predictor variables), the RRR-derived precipitation pattern score (PVS), and dietary pattern scores (response variables)**

| Predictor variables | | PVS | | Market-based diet (DP 1) | | Legume-based diet (DP 2) | | Vegetable-based diet (DP 3) | |
|---|---|---|---|---|---|---|---|---|---|
| | | **Unadj. model** | **Adj. model[a]** | **Unadj. model** | **Adj. model[a]** | **Unadj. model** | **Adj. model[a]** | **Unadj. model** | **Adj. model[a]** |
| CDDws (days) | Consecutive dry days in wet season | 0.70*** | 0.71*** | 0.20*** | 0.21*** | 0.12*** | 0.14*** | 0.40*** | 0.41*** |
| R10 (days) | Days with heavy precipitation | -0.57*** | -0.59*** | -0.10*** | -0.12*** | -0.07* | -0.10*** | -0.39*** | -0.40*** |
| CDD (days) | Consecutive dry days | -0.44*** | -0.58*** | -0.11*** | -0.19*** | -0.12** | -0.16*** | -0.26*** | -0.26*** |
| PRCPAUG (mm) | Cumulative rainfall in August | -0.60*** | -0.58*** | -0.21*** | -0.17*** | -0.12*** | -0.12*** | -0.31*** | -0.34*** |
| R99p (mm) | Extremely wet days | 0.55*** | 0.55*** | 0.19*** | 0.19*** | 0 | 0.01 | 0.35*** | 0.35*** |
| R20Aug (days) | Days with "big rains" in August | -0.54*** | -0.53*** | -0.18*** | -0.17*** | -0.04 | -0.05* | -0.33*** | -0.34*** |
| CDD (days) | Consecutive dry days | -0.40*** | -0.52*** | 0.01 | -0.07** | -0.07* | -0.13*** | -0.31*** | -0.34*** |
| PRCPJUL (mm) | Total wet-day precipitation in July | 0.55*** | 0.52*** | 0.18*** | 0.13*** | 0.06* | 0.05 | 0.32*** | 0.35*** |
| CWD (days) | Consecutive wet days | -0.39*** | -0.40*** | -0.06* | -0.07** | -0.05 | -0.06* | -0.27*** | -0.27*** |
| PRCPTOT (mm) | Annual total wet-day precipitation | -0.40*** | -0.37*** | -0.09*** | -0.07* | -0.11*** | -0.12*** | -0.22*** | -0.23*** |
| R20 (days) | Days with very heavy precipitation | -0.34*** | -0.36*** | -0.02 | -0.05 | -0.05 | -0.09** | -0.25*** | -0.26*** |
| R25 (days) | Days with very heavy precipitation | -0.34*** | -0.33*** | -0.06* | -0.05* | -0.11*** | -0.13*** | -0.18*** | -0.19*** |
| SDII (mm/day) | Simple daily intensity index | -0.28*** | -0.32*** | -0.07** | -0.10*** | -0.19*** | -0.22*** | -0.09** | -0.09** |
| R20Jul (days) | Days with "big rains" in July | 0.21*** | 0.18*** | 0.11*** | 0.08** | 0.06* | 0.04 | 0.08** | 0.08** |
| Lws (days) | Duration wet season | 0.16*** | 0.16*** | 0.08** | 0.08** | 0.01 | 0.01 | 0.08** | 0.08** |
| R95p (mm) | Very wet days | 0 | 0.09*** | -0.07** | 0.02 | -0.05 | 0.01 | 0.07** | 0.07** |
| **Total** | | | | **0.32*** ** | **0.29*** ** | **0.28*** ** | **0.28*** ** | **0.50*** ** | **0.51*** ** |

[a] Adjusted for child's age and sex, and stratum; Note: Correlations are considered weak 0 to 0.30, moderate 0.31 to 0.50, and strong 0.51 to 1.00

* p-value < 0.05; ** p-value < 0.01; *** p-value < 0.001
